# *Mycobacterium smegmatis* DinB2 misincorporates deoxyribonucleotides and ribonucleotides during templated synthesis and lesion bypass

**DOI:** 10.1093/nar/gku1027

**Published:** 2014-10-28

**Authors:** Heather Ordonez, Stewart Shuman

**Affiliations:** Molecular Biology Program, Sloan-Kettering Institute, New York, NY 10065, USA

## Abstract

*Mycobacterium smegmatis* DinB2 is the founder of a clade of Y-family DNA polymerase that is naturally adept at utilizing rNTPs or dNTPs as substrates. Here we investigate the fidelity and lesion bypass capacity of DinB2. We report that DinB2 is an unfaithful DNA and RNA polymerase with a distinctive signature for misincorporation of dNMPs, rNMPs and oxoguanine nucleotides during templated synthesis *in vitro*. DinB2 has a broader mutagenic spectrum with manganese than magnesium, though low ratios of manganese to magnesium suffice to switch DinB2 to its more mutagenic mode. DinB2 discrimination against incorrect dNTPs in magnesium is primarily at the level of substrate binding affinity, rather than *k*_pol_. DinB2 can incorporate any dNMP or rNMP opposite oxo-dG in the template strand with manganese as cofactor, with a kinetic preference for synthesis of an A:oxo-dG Hoogsteen pair. With magnesium, DinB2 is adept at synthesizing A:oxo-dG or C:oxo-dG pairs. DinB2 effectively incorporates deoxyribonucleotides, but not ribonucleotides, opposite an abasic site, with kinetic preference for dATP as the substrate. We speculate that DinB2 might contribute to mycobacterial mutagenesis, oxidative stress and quiescence, and discuss the genetic challenges to linking the polymerase biochemistry to an *in vivo* phenotype.

## INTRODUCTION

We are interested in the large and distinctive roster of DNA repair enzymes of the human pathogen *Mycobacterium tuberculosis* and its avirulent relative *Mycobacterium smegmatis.* These two mycobacterial species share a complement of eight DNA polymerases, of which one belongs to the A family (PolA) (1–4), two to the C family (DnaE1 and DnaE2) (5,6), three to the AEP family (LigD-POL, PolD1 and PolD2) (7–9) and two to the Y family (DinB1 and DinB2) (10–13). *M. smegmatis* encodes a third Y-family polymerase, DinB3, not present in *M. tuberculosis* (13).

DinB1 and DinB3 are template-dependent DNA polymerases that discriminate strongly against ribonucleotide substrates, a property that, in the case of DinB1, correlates with its aromatic steric gate side chain Phe23 (13). The steric gate of replicative and repair polymerases guards the genome against embedded ribonucleotides by clashing with the 2′-OH of an rNTP substrate. This residue is a phenylalanine or tyrosine for the B and Y family polymerases (14). Many studies have verified that subtraction of the aromatic steric gate elicits a gain of function in ribonucleotide utilization (14–18). We extended this theme to *M. smegmatis* DinB1 by showing that changing Phe23 to leucine conferred RNA polymerase activity (13).

DinB2 is the founder of a novel clade of Y-family polymerase that naturally lacks an aromatic steric gate and is adept at incorporating ribonucleotides. DinB2's sugar selectivity factor, determined from the rates (*k*_pol_) of dNMP versus rNMP incorporation at saturating dNTP/rNTP substrate concentrations, is 2.7- to 3.8-fold in manganese and 2.6- to 6-fold in magnesium (13). The vigorous RNA polymerase activity of mycobacterial DinB2 is governed by a leucine side chain in lieu of an aromatic steric gate. We speculated that the unique ability of DinB2 to utilize rNTPs might allow for DNA repair with a ‘ribo patch’ when dNTPs are limiting. Phylogenetic analysis revealed DinB2-like polymerases, with leucine, isoleucine or valine steric gates, in many taxa of the phylum *Actinobacteria* (13).

Genetic analyses of the *M. tuberculosis* paralogs DinB1 and DinB2 (10) highlighted stark differences in their regulation and biology *vis à vis Escherichia coli* DinB, also known as DNA polymerase IV (19). *E. coli* DinB is implicated in spontaneous and adaptive mutagenesis (19–22) and in bypassing bulky adducts such as N^2^-furfuryl-dG and alkylated purines 3-methyl-dA, and 3-methyl-dG (23–25). *E. coli dinB* is under the transcriptional control of the LexA repressor and is induced in response to DNA damage. By contrast, mycobacterial *dinB1* and *dinB2* are expressed constitutively during logarithmic growth and stationary phase and are neither induced by DNA damage nor dependent on RecA and SOS-response (10). *M. tuberculosis dinB1* and *dinB2* deletions, singly or together, have no effect on bacterial growth or sensitivity to nitrofurazone, nitroquinolone oxide, EMS, MMS or mitomycin C. There was no effect of *dinB1* and *dinB2* deletions on the rate of spontaneous mutation to rifampin resistance (10).

These findings prompt us to interrogate more deeply the biochemical properties of DinB2, with an eye toward gauging its potential contributions to mutagenesis and lesion bypass. In the present study, we focus on the following questions. How does the rate of incorporation of the correctly base-paired nucleotide compare to the rates of misincorporation of incorrect nucleotides? Is DinB2 fidelity influenced by whether the incoming substrate is a ribonucleotide or a deoxyribonucleotide? Is DinB2 fidelity affected by the use of magnesium versus manganese as the metal cofactor? The results of this inquiry highlight DinB2 as a comparatively unfaithful DNA and RNA polymerase with a distinctive signature for misincorporation of dNMPs, rNMPs and oxoguanine nucleotides during templated synthesis *in vitro*.

We also address the effects of two kinds of template DNA lesions on DinB2 activity: an abasic site and an oxoguanine deoxynucleoside (oxo-dG). These lesions can elicit different responses from different DNA polymerases (26,27), ranging from elongation arrest to bypass of the lesion, with or without mutagenesis. We report that DinB2 can incorporate any dNMP or rNMP opposite oxo-dG in the template strand with manganese as cofactor, with a kinetic preference for synthesis of an A:oxo-dG Hoogsteen pair. With magnesium as cofactor, DinB2 is adept at synthesis of either A:oxo-dG or C:oxo-dG configurations. DinB2 effectively incorporates deoxynucleotides, but not ribonucleotides, opposite an abasic site, with kinetic preference for dATP as the substrate.

## MATERIALS AND METHODS

### Materials

Recombinant DinB2 was produced in *E. coli* and purified as described previously (13). 8-oxo-dGTP and 8-oxo-GTP were purchased from TriLink BioTechnologies. DNA oligonucleotides, including those with chemical modifications (oxo-dG or a THF abasic nucleoside), were purchased from Eurofins MWG Operon.

### Primer-templates

The 5’ ^32^P-labeled primer DNA strand was prepared by reaction of a synthetic oligodeoxynucleotide with T4 polynucleotide kinase and [γ^32^P]ATP. The labeled DNA was heated to 95°C to inactivate the kinase. Primer DNA was then annealed to a 3-fold excess of a complementary DNA strand to form the primer-templates shown in the figures. The annealed DNAs were purified by electrophoresis through a native 15% polyacrylamide gel and then eluted from an excised gel slice by incubation for 16 h at 4°C in 300 μl of 10 mM Tris-HCl, pH 7.5, 1 mM EDTA, 50 mM NaCl.

### Polymerase assay

Reaction mixtures containing 10 mM Tris-HCl, pH 7.5, 50 nM 5′ ^32^P-labeled primer-template, and divalent cations, dNTPs or rNTPs and DinB2 as specified in the figure legends were incubated at 37°C. The reactions were initiated by adding DinB2 and quenched with an equal volume of 90% formamide, 50 mM EDTA, 0.3% bromophenol blue. The reaction products were analyzed by electrophoresis through a 15-cm 18% polyacrylamide gel containing 7 M urea in 89 mM Tris-borate, 2.5 mM EDTA. The products were visualized by autoradiography and, where indicated, quantified by scanning with a Fujix imager.

## RESULTS

### DinB2 misincorporates dNMPs and rNMPs with manganese as the metal cofactor

**Figure 1.**
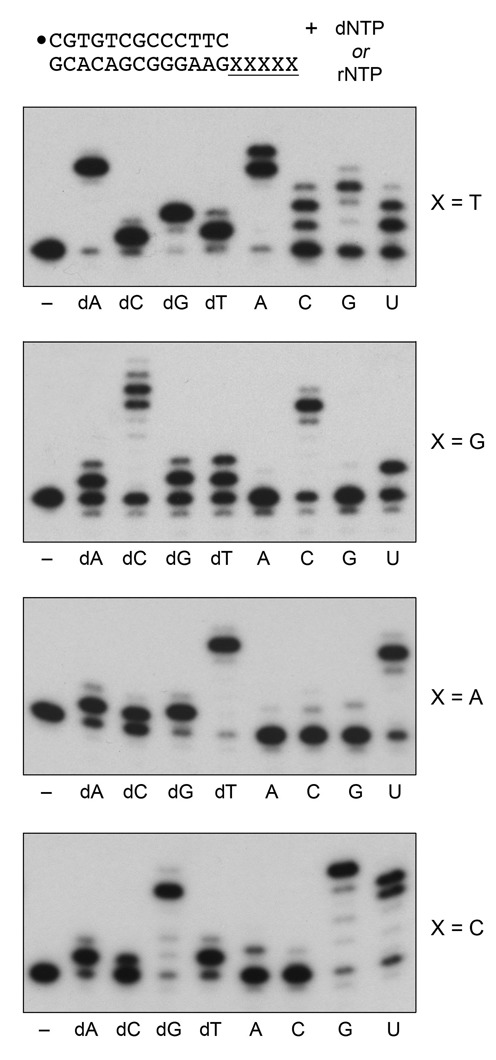
dNMP and rNMP misincorporation during Mn^2+^-dependent synthesis. Polymerase reaction mixtures (10 μl) containing 10 mM Tris-HCl, pH 7.5, 50 nM 5′ ^32^P-labeled 13-mer/18-mer primer-template as specified, 1 μM DinB2, 1 mM MnCl_2_, and either no nucleotide (–) or 100 μM of the indicated dNTP or rNTP were incubated at 37°C for 10 min. The reaction products were analyzed by urea-PAGE and visualized by autoradiography.

We exploited a set of 5′ ^32^P-labeled 13-mer/18-mer primer-templates consisting of an identical 13-bp duplex plus a five-nucleotide homo-oligomeric 5′ tail, either dA_5_, dC_5_, dG_5_ or dT_5_, that directs nucleotide addition to the primer strand. Polymerase reactions were performed in the presence of 1 mM Mn^2+^, 100 μM dNTP or rNTP substrate and a 20-fold DinB2 molar excess over primer-template. The ^32^P-labeled products generated during a 10 min reaction were analyzed by urea-PAGE and visualized by autoradiography. For the substrate with the oligo-dT_5_ template strand, DinB2 catalyzed five steps of dAMP addition to convert nearly all of the input primer to a single n+5 product of full fill-in synthesis (Figure 1, X = T). The instructive findings were that DinB2 catalyzed near-quantitative extension of the input primer by a single step of misincorporation of dCMP and dTMP or by two steps of misincorporation of dGMP. Thus, DinB2 is an unfaithful DNA polymerase that readily adds a mispaired dNMP opposite a template T, but does not readily extend with a second mispaired nucleotide from the mismatched C:T or T:T primer terminus thereby formed, or with a third mispaired dGMP opposite a tandem GG:TT mispaired primer end. With rNTP substrates, DinB2 efficiently extended the primer by four and five additions with the correctly templated AMP nucleotide. It also added, albeit with lower efficiency, one or two mispaired CMP and UMP nucleotides and three GMP nucleotides (Figure 1; X = T).

When programmed by the oligo-dG_5_ template strand, DinB2 extended the primer by six and seven additions with the correctly paired dCMP (Figure 1; X = G); apparently the oligo-dC product could slip back on the template dG tract and prime extra steps of dCMP addition. DinB2 misincorporated dAMP, dGMP and dTMP opposite G in the template, predominantly by one addition step, with a minority of primers receiving a second mispaired nucleotide. DinB2 added five correctly paired CMPs on the oligo-dG_5_ primer-template, but failed to misincorporate AMP or GMP opposite G (Figure 1; X = G), signifying higher fidelity as an RNA polymerase than a DNA polymerase for these particular N:G mispairs. On the other hand, DinB2 did misincorporate a single UMP opposite G.

The oligo-dA_5_ template directed DinB2 to extend the primer by five additions with the correctly paired dTMP nucleotide and by one addition with the mispaired dAMP, dCMP and dGMP nucleotides (Figure 1; X = A). In RNA polymerase mode, DinB2 faithfully added four correctly paired UMP nucleotides and had scant capacity to misincorporate AMP, CMP or GMP opposite a template A (Figure 1; X = A).

On the oligo-dC_5_ template, DinB2 catalyzed five additions with the correctly paired dGMP nucleotide and just one addition with the mispaired dAMP, dTMP and dCMP nucleotides (Figure 1; X = C). DinB2 added five correctly paired GMP nucleotides and three or four mispaired UMP nucleotides, but was loath to misincorporate AMP or CMP opposite C.

### Kinetics of addition of paired and mispaired dNTP and rNTP substrates

**Figure 2.**
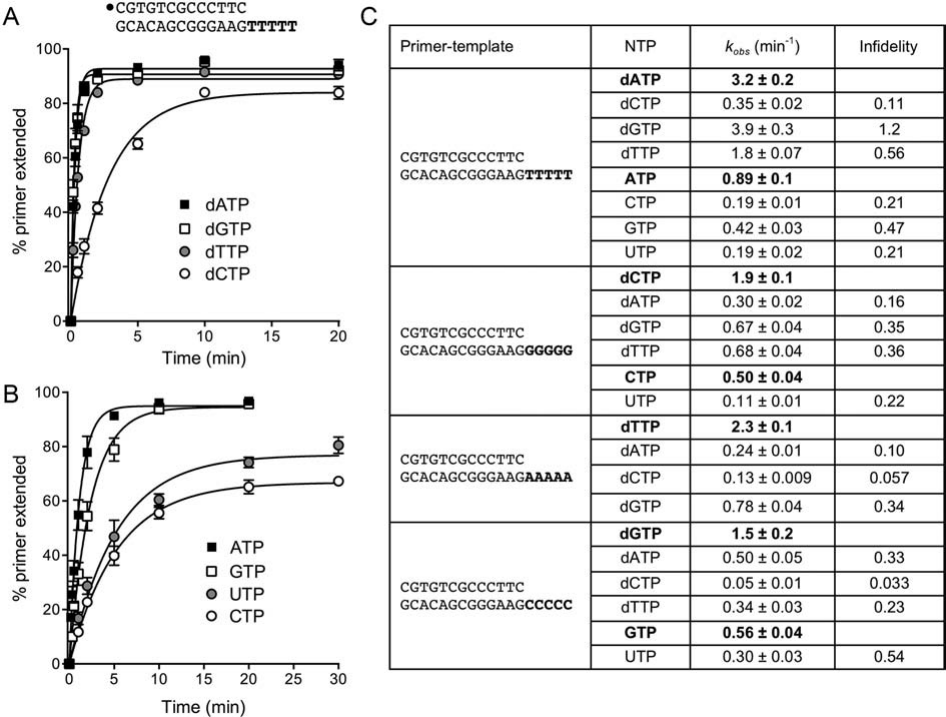
Kinetic analysis of DinB2 infidelity with manganese as the metal cofactor. (**A** and **B**) Polymerase reaction mixtures containing 10 mM Tris-HCl, pH 7.5, 50 nM 5′ ^32^P-labeled 13-mer/18-mer primer-template with an oligo-dT_5_ tail, 1 mM MnCl_2_, and either 100 μM deoxynucleoside triphosphate (panel A) or 100 μM ribonucleoside triphosphate (panel B) as specified were incubated at 37°C. Aliquots (10 μl) were withdrawn at the times specified and quenched immediately with EDTA/formamide. The reaction products were analyzed by urea-PAGE and the percent of primer strand extended by one or more nucleotides was quantified by scanning the gel with a Fujix BAS2500 imager. The % primer extension is plotted as a function of reaction time. Each datum in the graphs is the average of three separate experiments ±SEM. The rate constants (*k*_obs_ ± SE) for the first step of dNMP and rNMP addition to the primer-template were obtained by non-linear regression curve fitting of the data to a one-phase association function in Prism and are shown in panel **C**. A series of 5′ ^32^P-labeled 13-mer/18-mer primer-templates with identical duplex segments and either oligo-dG_5_, oligo-dA_5_ or oligo-dC_5_ template tails was used to assay the kinetics of nucleotide addition to the primer strand. The rate constants are compiled in panel C. Infidelity was calculated as the ratio of the rate of mispaired deoxynucleotide or ribonucleotide addition to the rate of addition of the correctly paired deoxynucleotide or ribonucleotide.

Exemplary kinetic data are shown in Figure 2 for DinB2-catalyzed primer extension on the oligo-dT_5_ template with each single dNTP substrate (panel A) or rNTP substrate (panel B). The percent of primer strand elongated by one or more steps is plotted as a function of time. By fitting the data to a one-phase association in Prism, we derived rate constants (*k*_obs_) for the first nucleotide addition step. The rates are compiled in Figure 2C. Infidelity is quantified in Figure 2C as the ratio of the rate of addition of each dNTP or rNTP versus that of the correctly templated substrate.

The results establish that DinB2 is an especially unfaithful DNA polymerase with a distinctive misincorporation signature. For example, DinB2 was faster at adding dG opposite T than it was at adding the correct dA nucleotide (infidelity = 1.2). Infidelity values of >0.3 were evident for the generation of five other mispairs: dT:dT (0.56); dT:dG (0.36); dG:dG (0.35); dG:dA (0.34) and dA:dC (0.33). The hierarchy of DinB2 infidelities for the remaining six mispairs was: dT:dC (0.23); dA:dG (0.16); dC:dT (0.11); dA:dA (0.1); dC:dA (0.057) and dC:dC (0.033). The salient theme is that DinB2 is most prone to misincorporate dGTP and dTTP and least prone to misincorporate dCTP.

DinB2 has a narrower spectrum of infidelity as an RNA polymerase, insofar as we were unable to derive rate constants for seven of the 12 rNTP:template-dX mispairs because DinB2 did not execute a sufficient level of rNMP addition during a 30 min reaction. In the cases where we were able to quantify rNMP misincorporation kinetics, the scale of infidelity was as follows: U:dC (0.54); G:dT (0.47); U:dG (0.22); U:dT (0.21) and C:dT (0.21). DinB2's ability to misincorporate U and G during RNA synthesis echoes its mismatch preferences in DNA synthesis.

### DinB2 infidelity with magnesium as metal cofactor

**Figure 3.**
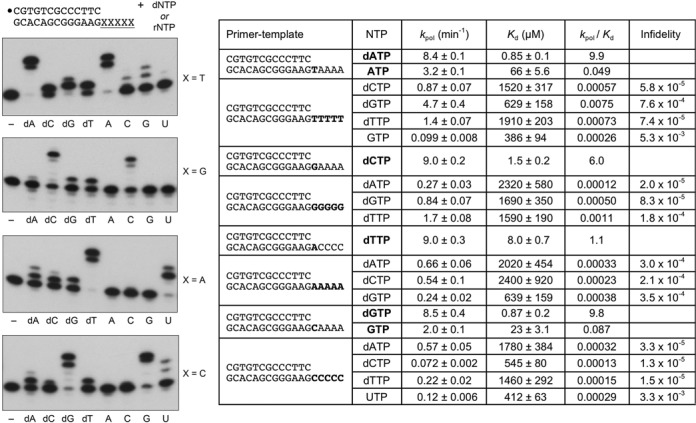
dNMP and rNMP misincorporation during magnesium-dependent synthesis. Left panel*.* Polymerase reaction mixtures (10 μl) containing 10 mM Tris-HCl, pH 7.5, 50 nM 5′ ^32^P-labeled 13-mer/18-mer primer-template as specified, 1 μM DinB2, 5 mM MgCl_2_ and either no nucleotide (–) or 500 μM of the indicated rNTP or dNTP were incubated at 37°C for 10 min. The reaction products were analyzed by urea-PAGE and visualized by autoradiography. Right panel. Polymerase reaction mixtures containing 10 mM Tris-HCl, pH 7.5, 5 mM MgCl_2_, 50 nM 5′ ^32^P-labeled 13-mer/18-mer primer-template, 1 μM DinB2, and varying concentrations of dNTP or rNTP substrates were incubated at 37°C. Aliquots (10 μl) were withdrawn at sequential timepoints and quenched with EDTA/formamide. The products were analyzed by urea-PAGE and quantified by scanning the gels. The % of primer extended by one or more nucleotides was plotted as a function of reaction time for each concentration of dNTP or rNTP substrate. The data were fit by non-linear regression in Prism to a one-phase exponential. The *k*_obs_ values calculated in Prism were plotted as a function of ATP concentration, with each datum being the average of two separate kinetic experiments. The data were fit by non-linear regression to a single binding function, from which the *K*_d_ for nucleotide and turnover number (*k*_pol_) at saturating nucleotide were derived. The *k*_pol_ and *K*_d_ values and *k*_pol_/*K*_d_ ratio (catalytic efficiency) for each dNTP and rNTP substrate are indicated. Infidelity was calculated as the ratio of the catalytic efficiency for the mispaired versus correctly paired deoxynucleotide and ribonucleotide substrates.

DinB2 efficiently scavenges limiting concentrations of dNTP and rNTP substrates in the presence of manganese (13), thereby confounding evaluation via single-turnover kinetics of the affinity of DinB2 for the correct incoming dNTP/rNTP substrate during manganese-dependent synthesis. In the presence of magnesium, we found that the rate of nucleotide addition displayed a classic dependence on the concentration of the first correctly templated dNTP or rNTP (13). The *k*_pol_ values for the correctly templated dNTPs were remarkably similar independent of the nucleobase (range 8.4 to 9.0 min^−1^) as were the *k*_pol_ values for the correctly templated rNTPs (range 1.5 to 3.2 min^−1^) (Figure 3). Whereas DinB2 displayed only a 2.6- to 6-fold differential in the rates of deoxy versus ribo addition at saturating nucleotide concentrations, the *K*_d_ for nucleotide substrate was strongly influenced by the nucleoside sugar. To wit, DinB2 has a 26- to 78-fold lower affinity for rNTPs than dNTPs (Figure 3).

Here, we surveyed misincorporation by DinB2 with magnesium as the metal cofactor. Polymerase reactions were performed in the presence of 5 mM Mg^2+^, 500 μM dNTP or rNTP substrate, and a 20-fold DinB2 molar excess over a 5′ ^32^P-labeled 13-mer/18-mer primer-template with oligo-dX_5_ template strand. Polyacrylamide gel electrophoresis (PAGE) analysis of the ^32^P-labeled products of a 10 min reaction is shown in Figure 3, left panels. With respect to its DNA polymerase activity, DinB2 catalyzed three to five steps of correctly templated dNMP addition in response to each oligo-dX_5_ template strand. Misincorporation was generally limited to a single step of mispaired dNMP addition. The oligo-dT_5_ template was the most conducive to misincorporation, with a dG:dT mispair being formed in near-quantitative yield.

Rate constants for dNMP misincorporation on each of the oligo-dX_5_ templates were determined at a series of increasing concentrations of dNTP substrate. By plotting *k*_obs_ as a function of [dNTP], and then fitting the data to a one-site binding site function, we derived an apparent affinity (*K*_d_) and a turnover number (*k*_pol_) for each dN:dX substrate:template mispair. The data are compiled in Figure 3, right panel, and are compared to the kinetic parameters for correct dNMP addition on the indicated primer-templates (13). Focusing first on *k*_pol_, we see that the maximum rates of incorrect dNMP addition span a wide range, from 4.7 min^−1^ for a dG:dT mispair to 0.072 min^−1^ for a dC:dC mispair. By dividing the mismatch *k*_pol_ values by *k*_pol_ for the correct dN:dX pair, we see that DinB2 displays the highest rates of magnesium-dependent misincorporation at dG:dT (infidelity ratio = 0.56), dT:dG (0.19) and dT:dT (0.17) mispairs. (These findings echo the rate hierarchy for manganese-dependent mispair synthesis; Figure 2.) At the other end of the spectrum, DinB2 was most faithful in its maximum rate of misincorporation at dC:dC (infidelity ratio = 0.008), dT:dC (0.026) and dG:dA (0.026) mispairs (Figure 3, right panel).

A salient finding from the kinetic analysis of magnesium-dependent misincorporation during DNA synthesis was that DinB2 discriminated strongly against incorrect dNTPs at the level of substrate binding affinity. Whereas *K*_d_ values for the correct dNTP varied from 0.85 to 8.0 μM (13), the affinities for incorrect dNTPs ranged from 545 to 2400 μM (Figure 3). When we derived *k*_pol_/*K*_d_ values as a measure of catalytic efficiency, and then gauged infidelity via the ratio of the *k*_pol_/*K*_d_ for mispair incorporation to the *k*_pol_/*K*_d_ for the correct dN:dX addition, we obtained infidelity values ranging from 1.3 × 10^−5^ (for dC:dC) to 7.6 × 10^−4^ (for dG:dT).

### Ribonucleotide misincorporation with magnesium as metal cofactor

DinB2 catalyzed two to four steps of correctly templated rNMP addition directed by the oligo-dX_5_ strands (Figure 3, left panel). Whereas low levels of misincorporation of one or two rNMPs were noted for the G:dT, C:dT and U:dC mispairs, there was no appreciable misincorporation at the other nine rN:dX mispairs during a 10 min reaction (Figure 3, left panel). We were able to determine *k*_pol_ and *K*_d_ values for two of the rN:dX mispairs and compare them to the kinetic parameters for addition of the correctly templated ribonucleotide (13). The *k*_pol_ for GMP insertion opposite dT (0.099 min^−1^) was 32-fold slower than correct AMP addition and the affinity for GTP (*K*_d_ 386 μM) was 5.8-fold lower than for ATP (*K*_d_ 66 μM), which translated into an infidelity value of 0.0053 (Figure 3). The *k*_pol_ for UMP insertion opposite dC (0.12 min^−1^) was 17-fold slower than correct GMP addition and the affinity for UTP (*K*_d_ 412 μM) was 18-fold lower than for GTP (*K*_d_ 23 μM), yielding an infidelity value of 0.0033 (Figure 3).

### Metal-dependent switch in DinB2 fidelity

**Figure 4.**
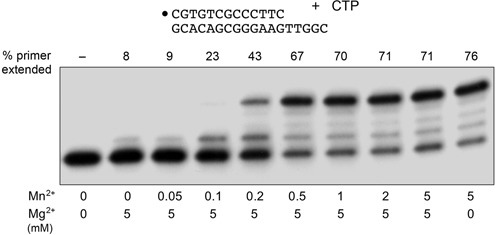
Metal-dependent switch in rC:dT mispair synthesis. Polymerase reaction mixtures (10 μl) containing 10 mM Tris-HCl, pH 7.5, 100 μM CTP, 50 nM 5′ ^32^P-labeled 13-mer/18-mer primer-template as shown, 1 μM DinB2, and MgCl_2_ or MnCl_2_ as specified were incubated at 37°C for 10 min. The reaction products were analyzed by urea-PAGE and visualized by autoradiography. The% of primer extended is indicated above the lanes.

Our experiments show that DinB2 has a broader spectrum of nucleotide misincorporation with manganese as cofactor than with magnesium (Figure 1 versus Figure 3). Although the intracellular concentrations of magnesium and manganese in *M. smegmatis* have not, to our knowledge, been reported, it is safe to assume that magnesium is in excess over manganese. This raises the pertinent question of how DinB2 behaves with respect to fidelity when both divalent cations are present. To address the issue, we conducted a metal mixing experiment, as shown in Figure 4, wherein we exploited the fact that DinB2 readily misincorporates CMP opposite dT in the presence of manganese but not in 5 mM magnesium. On the template 3′-TTGGC-5′ sequence used in Figure 4, manganese allowed extension of 76% of the input primer to yield a predominant n+4 product, reflecting two steps of CMP misincorporation opposite dT followed by two steps of correct CMP incorporation opposite dG. By contrast, magnesium permitted elongation of 8% of the primer by only a single addition step. The experiment entailed mixing 5 mM magnesium with increasing concentrations of manganese. We observed a manganese concentration-dependent binary switch in the product distribution, manifest as a steady increase in the % of primer extended and accumulation of the n+4 product, a transition that was virtually complete at 0.5 mM manganese, i.e. a 1:10 ratio of Mn^2+^ to Mg^2+^. By plotting the increase in primer extended as a function of [Mn^2+^] and fitting the data to a simple hyperbolic binding function, we obtained a *K*_d_ of 0.2 mM Mn^2+^ in effecting the switch from higher to lower fidelity in the presence of 5 mM magnesium. Thus, it appears that DinB2 has a preference for manganese occupancy of at least one of its two metal-binding sites (12,28) when both magnesium and manganese are present and that this occupancy suffices to shift DinB2 to a mutagenic misincorporation mode.

### Infidelity of DinB2 incorporation of oxoguanine nucleotides

**Figure 5.**
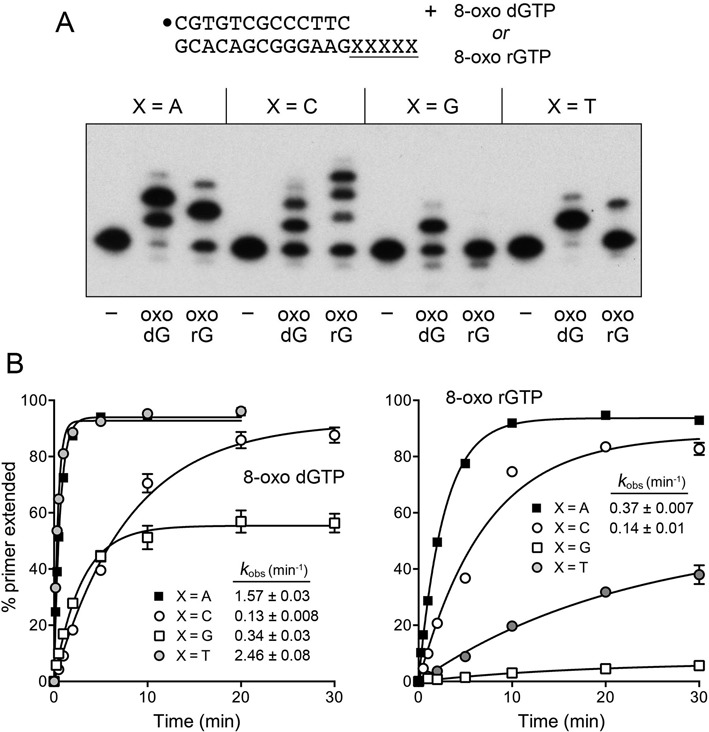
Infidelity of manganese-dependent 8-oxo-dGMP and 8-oxo-GMP incorporation. (**A**) Polymerase reaction mixtures containing 10 mM Tris-HCl, pH 7.5, 50 nM 5′ ^32^P-labeled 13-mer/18-mer primer-template as specified, 1 μM DinB2, 1 mM MnCl_2_, and either 100 μM oxo-dGTP or oxo-rGTP were incubated at 37°C for 10 min. The reaction products were analyzed by urea-PAGE and visualized by autoradiography. (**B**) Kinetics. Primer extension reactions were constituted as in (A). Aliquots were withdrawn at the times specified and quenched with EDTA/formamide. The % primer extension with oxo-dGTP (left panel) or oxo-rGTP (right panel) is plotted as a function of reaction time for each oligo-dX_5_ template. Each datum is the average of three separate experiments ±SEM. The rate constants (*k*_obs_ ± SE) for the first step of oxo-dGMP addition to the primer-template were obtained by non-linear regression curve fitting of the data to a one-phase association function in Prism.

Oxidative damage to guanine nucleoside triphosphates generates 8-oxo-dGTP and 8-oxo-rGTP that can serve as effective substrates for certain nucleic acid polymerases (29–31). oxo-dGTP in the dNTP pool is a threat to genomic integrity, because the oxidized guanine nucleobase in *syn* nucleoside conformation forms a Hoogsteen pair with adenine that can allow DNA polymerase to embed a mutagenic oxoG:A mispair during DNA synthesis. Here we examine the utilization of oxo-dGTP and oxo-rGTP (at 100 μM concentration) as substrates for DinB2-catalyzed primer extension on the homo-oligo-dX_5_ template strands in the presence of manganese (Figure 5A). In DNA synthesis mode, DinB2 extended virtually all of the input primer by oxo-dGMP addition opposite either a dA or dT template nucleotide, by one or two oxo-dG:dA addition steps or by one oxo-dG:dT addition step. DinB2 was conspicuously less active in adding oxo-dG opposite dC (the ‘correct’ paired template nucleobase) with respect to the percent of input primer extended by one or two oxo-dGMP steps. DinB2 was similarly less effective in a single step of oxo-dGMP addition opposite a dG template nucleotide (Figure 5A). The kinetic profiles for oxo-dGMP addition directed by the oligo-dX_5_ templates are shown in Figure 5B, with rate constants (*k*_obs_) derived by non-linear regression curve fitting of the data. The fastest rate observed was for oxo-dG:dT mispair synthesis (2.46 min^−1^), which was 63% of the rate of dG:dT mispair synthesis and 77% of the rate of synthesis of a correct dA:dT pair (Figure 2C), signifying that DinB2 barely discriminates oxidized versus unmodified guanine when forming its favorite dG:dT mispair. The rate of oxo-dG:dA mispair synthesis (or rather, Hoogsteen pair-directed synthesis) of 1.57 min^−1^ was 2-fold faster than the rate of dG:dA mispair synthesis and 68% of the rate of correctly paired dT:dA addition (Figure 2C). DinB2 was least fleet in synthesizing a ‘correct’ oxo-dG:dC pair, for which the observed rate (0.13 min^−1^) was 19-fold slower than oxo-dG:dT synthesis and 12-fold slower than a correctly paired dG:dC addition (Figures 5B and 2C). Thus, DinB2 is liberally mutagenic when provided with oxo-dGTP in the presence of manganese.

The kinetics of primer extension with an oxo-rGTP substrate are shown in Figure 5B and reveal a different misincorporation signature compared to oxo-dGTP, whereby DinB2 is most adept at oxo-rGMP addition opposite dA and dC and comparatively feeble at oxo-rGMP incorporation opposite dT and dG. We were able to derive rate constants for oxo-rG:dA (0.37 min^−1^) and oxo-rG:dC (0.14 min^−1^) additions. Whereas the oxo-rG:dA rate was one-fourth that of oxo-dG:dA misincorporation, DinB2 incorporated oxo-rGMP and oxo-dGMP at virtually the same rates opposite a dC template nucleotide (0.13 to 0.14 min^−1^). The vigorous activity of DinB2 in adding oxo-rG opposite dA contrasts with its ineffectiveness in forming a G:dA mispair (Figure 1). Although the extent of G:dA addition was too low to derive a rate constant, by comparing initial rates, we deemed oxo-rG:dA synthesis to be 40-fold faster than G:dA addition (data not shown), thereby attesting to the positive influence of the oxoG:A Hoogsteen pair on DinB2 ribonucleotide addition.

### Oxoguanine nucleotide misincorporation with magnesium as metal cofactor

**Figure 6.**
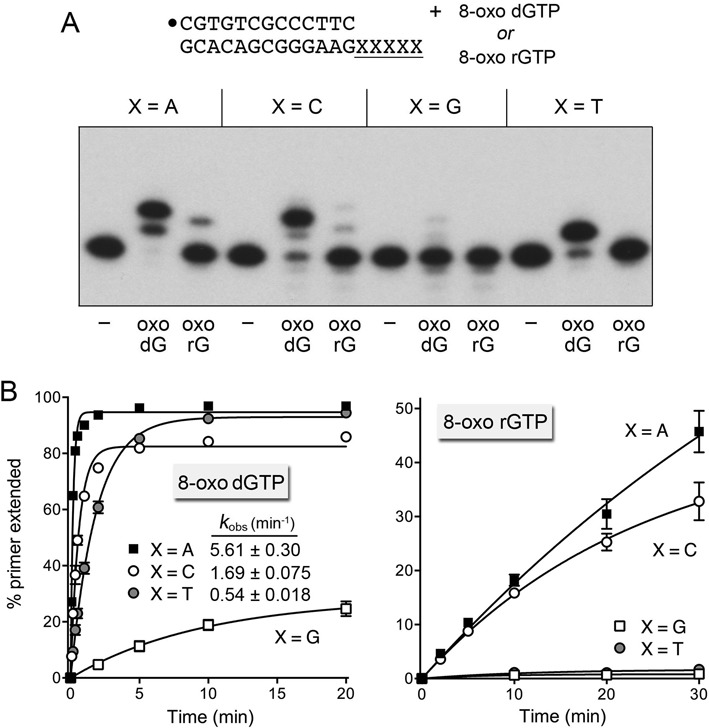
Infidelity of magnesium-dependent 8-oxo-dGMP and 8-oxo-GMP incorporation. (**A**) Polymerase reaction mixtures containing 10 mM Tris-HCl, pH 7.5, 50 nM 5′ ^32^P-labeled 13-mer/18-mer primer-template as specified, 1 μM DinB2, 5 mM MgCl_2_ and either 500 μM oxo-dGTP or oxo-rGTP were incubated at 37°C for 10 min. The reaction products were analyzed by urea-PAGE and visualized by autoradiography. (**B**) Kinetics. Primer extension reactions were constituted as in (A). The % primer extension with oxo-dGTP (left panel) or oxo-rGTP (right panel) is plotted as a function of reaction time for each oligo-dX_5_ template. Each datum is the average of three separate experiments ±SEM. The rate constants (*k*_obs_ ± SE) for oxo-dGMP addition are indicated.

The products of a DinB2-catalyzed primer extension reaction on the homo-oligo-dX_5_ template strands in the presence of 5 mM magnesium and either oxo-dGTP or oxo-rGTP (at 500 μM concentration) are shown in Figure 6A. DinB2 extended most of the input primer by two steps of oxo-dGMP addition opposite either a dA or dC template nucleotide, and by one oxo-dGMP step opposite a dT template nucleotide. By contrast, there was scant incorporation of oxo-dGMP on the oligo-dG_5_ template. The kinetic profiles for magnesium-dependent DNA synthesis with oxo-dGTP substrate are shown in Figure 6B. The fastest rate was for oxo-dG:dA mispair synthesis (*k*_obs_ 5.61 min^−1^), which was 23-fold greater than the *k*_pol_ value of 0.24 min^−1^ for dG:dA mispair synthesis (attesting to the salutary effect of Hoogsteen pairing) and 62% of the *k*_pol_ value of 9.0 min^−1^ for synthesis of a correct dT:dA pair (Figure 3). The rate of correctly paired oxo-dG:dC synthesis (*k*_obs_ 1.69 min^−1^) was 30% that of the oxo-dG:dA Hoogsteen pair and 20% of the *k*_pol_ of 8.5 min^−1^ for a dG:dC addition (Figure 3). The *k*_obs_ of 0.54 min^−1^ for oxo-dG:dT addition was 11% of the *k*_pol_ of 4.7 min^−1^ for synthesis of a dG:dT mispair. It is noteworthy that the *k*_obs_ values for the oxo-dG:dA and oxo-dG:dC additions were greater in magnesium than manganese (by 3.6-fold for oxo-dG:dA and 13-fold for oxo-dG:dC); by contrast, *k*_obs_ for oxo-dG:dT addition was 4.6-fold greater in manganese than magnesium. The rate and extent of oxo-dG:dG addition in magnesium was comparatively feeble; by comparing initial rates, we deemed the favored oxo-dG:dA synthesis step to be 140-fold faster than oxo-dG:dG addition.

DinB2 was able to add oxo-rG opposite dA and dC template nucleotides in the presence of magnesium; however, the initial rates of oxo-rG:dA and oxo-rG:dC synthesis were 160-fold and 66-fold slower than the corresponding oxo-dG additions (Figure 6B). DinB2 was unable to incorporate oxo-rG opposite dG or dT in the template strand. In general, DinB2 was much less effective in utilizing oxo-rGTP as a substrate with magnesium as cofactor than it was with manganese.

### Infidelity of nucleotide addition opposite an oxo-dG lesion in the template strand

**Figure 7.**
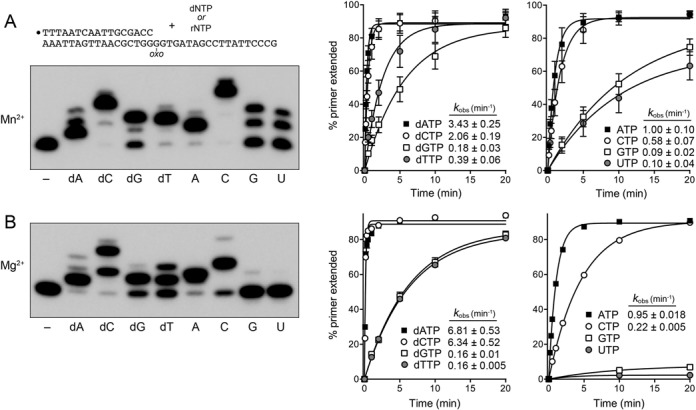
Nucleotide addition opposite oxo-dG in the template strand. Polymerase reaction mixtures containing 10 mM Tris-HCl, pH 7.5, 50 nM 5′ ^32^P-labeled 17-mer/36-mer primer-template as shown, 1 μM DinB2 and either 1 mM MnCl_2_ and 100 μM of the indicated dNTP or rNTP (**A**), or 5 mM MgCl_2_ and 500 μM of the indicated dNTP or rNTP (**B**) were incubated at 37°C for 10 min. Reaction mixtures for kinetic experiments were constituted as in (A) and (B). The % primer extension with dNTPs (left) or rNTPs (right) is plotted as a function of time in the presence of either MnCl_2_ (top) or MgCl_2_ (bottom). The rate constants (*k*_obs_ ± SE) for dNMP or rNMP addition are indicated.

A 5′ ^32^P-labeled 17-mer/36mer primer-template with a single oxo-dG lesion in the template strand immediately following the primer 3′-OH terminus (Figure 7A) was used to study the ability of DinB2 to insert deoxynucleotides and ribonucleotides opposite the oxo-dG nucleobase. With manganese as cofactor, DinB2 extended virtually all of the input primer by four steps of dCMP addition, entailing: (i) initial formation of a ‘correct’ dC:oxo-dG pair; (ii) correctly templated synthesis of the next dC:dG pair; (iii) synthesis of a dC:dT mispair, either directly or by primer slippage on the template GGGG tract and (iv) correctly templated synthesis of the next dC:dG pair (Figure 7A). A kinetic analysis revealed a rate constant of 2.06 min^−1^ for dCMP incorporation opposite oxo-dG; this value was similar to *k*_obs_ of 1.9 min^−1^ for dCMP addition opposite dG, signifying that DinB2 is unaffected by the 8-oxo lesion when it comes to faithful synthesis of a Watson–Crick pair with the incoming dCTP substrate. In the presence of dATP, DinB2 extended all of the primer, albeit predominantly by adding a single dAMP opposite oxo-dG (Figure 7A). The rate of dA:oxo-dG synthesis (*k*_obs_ 3.43 min^−1^) was 66% greater than that of the dC:oxo-dG addition, and was 11-fold greater than dA:dG mispair synthesis (0.30 min^−1^; Figure 2C), again testifying to the positive contribution of the Hoogsteen pairing. With dTTP, DinB2 converted virtually all of the input primer to an n+2 extension product, via synthesis of a dT:oxo-dG mispair, followed by favored synthesis of a dT:dG mispair. The rate of synthesis of the dT:oxo-dG mispair (*k*_obs_ 0.39 min^−1^) was 11% that of dA:oxo-dG synthesis and 57% that of dT:dG mispair synthesis (0.68 min^−1^; Figure 2C). With dGTP as substrate, DinB2 synthesized predominantly n+2 product (Figure 7A). The rate of dG:oxo-dG mispair synthesis was the slowest of the lot, at 0.18 min^−1^, which was 27% the rate of synthesis of a dG:dG mispair. In sum, DinB2 was capable of efficiently (with respect to yield) incorporating any dNMP opposite a template oxo-dG with manganese as cofactor, while kinetically favoring the mutagenic dA:oxo-dG addition.

Similar trends were observed for manganese-dependent ribonucleotide addition opposite an oxo-dG template lesion, whereby DinB2 converted all of the primer to an n+4 product in the presence of CTP and to an n+1 product in the presence of ATP. The rate constant for A:oxo-dG synthesis (1.0 min^−1^) was 72% greater than for the Watson–Crick C:oxoG pair (0.58 min^−1^). GTP (0.09 min^−1^) and UTP (0.10 min^−1^) were the less favored substrates for incorporation by DinB2 opposite oxo-dG.

The products and rates of DinB2 synthesis opposite oxo-dG with magnesium as cofactor are shown in Figure 7B. The *k*_obs_ of 6.81 min^−1^ for dA:oxo-dG formation (predominantly a single step of dAMP addition to the primer) was 80% of the *k*_pol_ value for a correct dA:dT pair and 25-fold greater than *k*_pol_ for a dA:dG mispair. The *k*_obs_ for synthesis of the Watson–Crick dC:oxo-dG pair was 6.34 min^−1^. Note that the products of extension with dCTP were a mixture of n+2 and n+4 species, suggesting that DinB2 was more prone in magnesium than manganese to pause after adding two G-templated dCMP nucleotides, at the first encounter with a dT template base. dGMP and dTMP were incorporated comparatively slowly (0.16 min^−1^) opposite oxo-dG. In RNA polymerase mode with magnesium as cofactor, DinB2 was effective at incorporating AMP (one step) and CMP (two steps, with very scant n+4 product) opposite oxo-dG, in high yield with respect to primer utilization (Figure 7B). ATP was the kinetically favored substrate (*k*_obs_ 0.95 min^−1^) versus CTP (0.22 min^−1^). DinB2 was unable to utilize GTP or UTP as substrates for magnesium-dependent rNMP addition opposite oxo-dG.

### Nucleotide addition opposite an abasic lesion in the template strand

**Figure 8.**
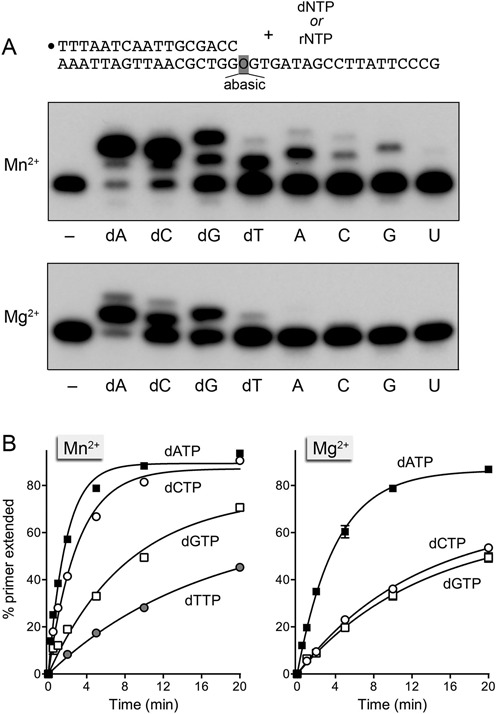
Nucleotide addition opposite an abasic site in the template strand. (**A**) Polymerase reaction mixtures containing 10 mM Tris-HCl, pH 7.5, 50 nM 5′ ^32^P-labeled 17-mer/36-mer primer-template as shown, 1 μM DinB2, and either 1 mM MnCl_2_ and 100 μM of the indicated dNTP or rNTP (top panel), or 5 mM MgCl_2_ and 500 μM of the indicated dNTP or rNTP (bottom panel) were incubated at 37°C for 10 min. (**B**) Kinetics. Primer extension reactions were constituted as in (A). The % primer extension with each dNTP in the presence of MnCl_2_ (left) or MgCl_2_ (right) is plotted as a function of reaction time. Each datum is the average of three separate experiments ± SEM.

A 5′ ^32^P-labeled 17-mer/36mer primer-template with a single THF (tetrahydrofuran) abasic site in the template strand immediately following the primer 3′-OH terminus (Figure 8A) was used to study the ability of DinB2 to insert deoxynucleotides and ribonucleotides without immediate instruction from a templating nucleobase. With manganese as cofactor, DinB2 extended most of the input primer by two steps of dAMP or dCMP addition. Albeit with lower yield, DinB2 also extended the primer by one and two steps of dGMP addition and by one round of dTMP addition (Figure 8A). Kinetic analysis revealed rate constants for dNMP incorporation opposite the abasic site as follows: 0.54 ± 0.025 min^−1^ for dAMP; 0.33 ± 0.019 min^−1^ for dCMP, 0.12 ± 0.015 min^−1^ for dGMP and 0.061 ± 0.009 min^−1^ for dTMP (Figure 8B, left panel). The *k*_obs_ for dAMP addition at the abasic site was 17% of *k*_obs_ for templated synthesis of a correct dA:dT pair (3.2 min^−1^; Figure 2C). Although DinB2 was relatively feeble in its ability to incorporate ribonucleotides opposite the abasic site, to the extent that it did, it preferred to use ATP over CTP and GTP, with UTP being ineffective (Figure 8A). The initial rate of AMP addition was 15-fold slower than that of dAMP addition (data not shown). The initial rates of CMP and GMP addition were one-third that of AMP (not shown).

With magnesium as cofactor, DinB2 extended most of the input primer by one step of dAMP addition. The yield of extended primer was lower with dCTP and dGTP substrates, whereas dTTP was ineffective (Figure 8A). Observed rates constants for dNMP incorporation opposite the abasic site were 0.25 ± 0.01 min^−1^ for dAMP, 0.08 ± 0.006 min^−1^ for dCMP and 0.08 ± 0.01 min^−1^ for dGMP (Figure 8B, right panel). DinB2 was unable to utilize rNTPs as substrates for magnesium-dependent addition opposite an abasic template lesion (Figure 8A). Our results indicate that DinB2, like many other DNA and RNA polymerases (26,32–34), obeys an ‘A-rule’ whereby it prefers to incorporate an adenine nucleotide opposite an abasic site in the DNA template.

## DISCUSSION

Our biochemical analyses of DinB2 polymerase are broadly pertinent to three interrelated themes in mycobacterial physiology: mutagenesis, oxidative stress and quiescence. We discuss our findings as they relate to these themes and attempt to draw connections between them, while highlighting the challenges ahead to linking the polymerase biochemistry to an *in vivo* readout.

Polymerase fidelity is a central issue in mycobacterial mutagenesis and is relevant to public health insofar as mycobacterial chromosomal mutations underlie virtually all clinical resistance to anti-tuberculosis drugs. Prior studies implicated DnaE2 as a major agent of damage-induced mutagenesis and drug resistance (5), but the biochemical properties of DnaE2 that underpin its pro-mutagenic role are *tabula rasa*. The situation is inverted for DinB2, insofar as the available genetics are unrevealing with respect to a phenotype (10), but the biochemical information is now substantial. Via a kinetic analysis of the fidelity of the DinB2 DNA polymerase in copying an undamaged DNA template, we find that: (i) DinB2 is a mutagenic polymerase with a distinctive error signature, whereby DinB2 is most prone to misincorporate dGTP and dTTP and least adept at misincorporating dCTP; (ii) DinB2 has a broader mutagenic spectrum with manganese as cofactor than with magnesium; (iii) low ratios of manganese to magnesium suffice to switch DinB2 to its more mutagenic mode and (iv) DinB2 discrimination against incorrect dNTPs in the presence of magnesium is primarily at the level of substrate binding affinity, rather than *k*_pol_. Prior studies showed that ablation of *dinB2* had little effect on the spontaneous mutation rate of *M. tuberculosis* to rifampin resistance, but did alter the spectrum of base mutations within the three codons in *rpoB* that confer drug resistance (10). The knowledge gained here regarding DinB2's misincorporation preferences *in vitro* could aid in the design of more sensitive genetic reporters of DinB2's pro-mutagenic activity *in vivo*.

Mycobacteria encounter and respond to oxidative stress (35,36). One consequence of oxidative stress is the generation of oxoguanine nucleoside triphosphate precursors for nucleic acid synthesis and of oxo-dG lesions in chromosomal DNA. Oxidation of the guanine nucleotide pool is thought to underlie cell death triggered by bactericidal antibiotics (37). In *E. coli*, overexpression of the DinB polymerase is lethal, by virtue of its utilization of oxoguanine nucleotide substrates (37). Here we provide a detailed description of the ability of DinB2 to utilize oxo-dGTP as a substrate for DNA synthesis and the extremely pro-mutagenic capacity of DinB2 to incorporate oxo-dGMP opposite any template nucleobase. DinB2 easily synthesizes an oxo-dG:dA Hoogsteen pair, and it is much better at incorporating oxo-dG opposite dA than dC. Most impressive (to us) was the facility of DinB2 in forming an oxo-dG:dT mispair, at a rate even faster (in manganese) than synthesis of oxo-dG:dA. We conclude that the dG:dT mispair is a strong mutagenic signature of DinB2, whether or not the guanine base of the dNTP substrate is oxidized. Probing how DinB2 handles an oxo-dG lesion in the template strand revealed similar promiscuity, whereby any incoming dNTP could be polymerized opposite oxo-dG, albeit with kinetic preference for the A:oxo-dG Hoogsteen pair.

These findings raise the question of whether DinB2 might mediate or exacerbate the effects of oxidative stress on mycobacteria, by embedding oxidized nucleotides during DNA repair and creating mutations *en route*, or when copying an oxidized template. In the same vein, might DinB2 contribute to antibiotic sensitivity in mycobacteria, as seen in *E. coli* (37)? Answering these questions genetically may not be straightforward, insofar as mycobacteria have multiple MutT enzymes that detoxify oxo-dGTP in the NTP pool, by converting it to oxo-dGMP (38–40). Mycobacteria also have a MutM glycosylase that excises the oxoG nucleobase at an oxo-dG:dC pair and a MutY glycosylase that excises the dA nucleobase at an oxo-dG:dA pair (41,42). These cleansing systems might mask the potential of DinB2 (or other polymerases) to create oxidative damage and mutations. In that case, uncovering a *dinB2* deletion or overexpression phenotype might require the construction of mycobacterial strains multiply deleted for all of the enzymes that detoxify the oxo-dGTP pool and rectify oxo-dG sites in DNA.

Quiescence is central to the long-term carriage of *M. tuberculosis* in a clinically dormant state. Quiescent cells that are not replicating their DNA are generally thought to have reduced dNTP pools compared to actively dividing cells, resulting in a high rNTP:dNTP ratio in the polymerase substrate pool. Whereas, to our knowledge, the intracellular concentrations of dNTPs and rNTPs in mycobacteria have not been reported for any growth conditions, we assume that rNTPs prevail in non-replicating mycobacteria. We have suggested that DNA repair with a ‘ribo patch’ by polymerase utilization of available rNTPs is an intelligent strategy for quiescent cells to avoid otherwise deadly chromosome damage. DinB2 is one of four mycobacterial DNA polymerases that readily incorporate ribonucleotides during primer extension and gap repair *in vitro* (9,13). DinB2 is unique among them in its ability to synthesize longer RNA tracts on a DNA template strand (13).

There has been a recent surge of interest in the biological impact of ribonucleotides embedded in DNA. Many studies, primarily in eukaryal model systems, have shown that persistent ribonucleotides in genomic DNA are pro-mutagenic and that there are distinct pathways of ribonucleotide surveillance and ribonucleotide excision repair that deal with these potentially harmful ‘lesions’ (reviewed in (43)). The prevailing view is that polymerases are designed with steric gates to avoid ribonucleotide incorporation, and that the presence of embedded ribonucleotides reflects the intrinsic error rate in polymerase sugar selectivity. Indeed, many studies of embedded ribonucleotides take advantage of DNA polymerase mutants that have reduced sugar discrimination in order to force higher, non-physiological levels of ribonucleotide addition during DNA synthesis *in vivo*. The persistence (and hence impact) of ribonucleotides in DNA is further amplified by eliminating RNase H2, which incises the 5’-phosphodiester of the embedded ribonucleotide to initiate the pathway of ribonucleotide excision repair (43). Similar strategies have illuminated ribonucleotide excision pathways in *E. coli* and *Bacillus subtilis* (44–46).

DinB2 is distinctive among Pol IV enzymes in being naturally adept as an RNA polymerase (13). Here we show that DinB2 is an unfaithful RNA polymerase that readily creates rN:dX mispairs on an undamaged DNA template, particularly when using manganese as the metal cofactor. DinB2's preference for misincorporating U and G during RNA synthesis resembles its mutagenic signature in DNA synthesis. The mutagenic character of DinB2's RNA polymerase is greatly enhanced (with either manganese or magnesium) when using oxo-rGTP as a substrate for polymerization or when copying with standard rNTPs across an oxo-dG lesion in the template DNA strand. Thus, DinB2 emerges as a potential embedder of mispaired ribonucleotides in chromosomal DNA *in vivo*, especially under conditions of oxidative stress. Embedded mispaired ribonucleotides are mutagenic *in vivo* in *E. coli* in the absence of RNase H enzymes (47). The *in vivo* impact of DinB2's RNA polymerase activity is undoubtedly blunted, if not obscured, by the presence in the *M. smegmatis* proteome of four different RNase H enzymes: MSMEG_5562/RnhA (48); MSMEG_4305 (49); MSMEG_2442/RnhB and MSMEG_5849 (50). Unveiling the prevalence of ribonucleotides in mycobacterial genomic DNA and the roles of DinB2 and other polymerases in their embedding will require the systematic deletion of the genes encoding the RNase H enzymes, singly and in all viable combinations.
